# Microalgae Photo-Protectants and Related Bio-Carriers Loaded with Bioactive Entities for Skin Applications—An Insight of Microalgae Biotechnology

**DOI:** 10.3390/md20080487

**Published:** 2022-07-28

**Authors:** Berenice Santiesteban-Romero, Manuel Martínez-Ruiz, Juan Eduardo Sosa-Hernández, Roberto Parra-Saldívar, Hafiz M. N. Iqbal

**Affiliations:** 1 Tecnologico de Monterrey, School of Engineering and Sciences, Monterrey 64849, Mexico; a01113721@tec.mx (B.S.-R.); mmruiz@tec.mx (M.M.-R.); eduardo.sosa@tec.mx (J.E.S.-H.); 2 Tecnologico de Monterrey, Institute of Advanced Materials for Sustainable Manufacturing, Monterrey 64849, Mexico

**Keywords:** microalgae, photo protectants, sporopollenin, scytonemin, MAAs, bio-carriers, biomedical application

## Abstract

Microalgae are photosynthetic organisms known for producing valuable metabolites under different conditions such as extreme temperatures, high salinity, osmotic pressure, and ultraviolet radiation. In recent years, these metabolites have become a trend due to their versatility in applications such as pharmaceuticals, cosmetics, and others. They have even been proposed as an alternative source of bioactive metabolites to avoid the harmful effects on the environment produced by active compounds such as oxybenzone in commercials sunscreens. One of the most studied applications is the use of microalgae for skin care and topical use as cosmeceuticals. With the increasing demand for more environmentally friendly products in cosmetics, microalgae have been further explored in relation to this application. It has been shown that some microalgae are resistant to UV rays due to certain compounds such as mycosporine-like amino acids, sporopollenin, scytonemin, and others. These compounds have different mechanisms of action to mitigate UV damage induced. Still, they all have been proven to confer UV tolerance to microalgae with an absorbance spectrum like the one in conventional sunscreens. This review focuses on the use of these microalgae compounds obtained by UV stimulation and takes advantage of their natural UV-resistant characteristics to potentially apply them as an alternative for UV protection products.

## 1. Introduction

**Figure 1:**
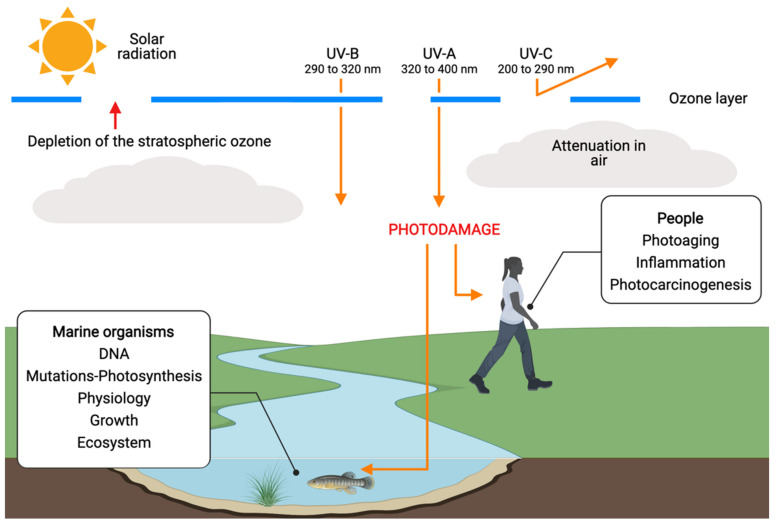
Negative effects of different UV rays on people, including photoaging, inflammation and the possible occurrence of skin cancer. In marine organisms, UV rays affect as well as DNA and organism growth. Created with BioRender.com (accessed on 1 May 2022) and extracted under premium membership.

The increased solar ultraviolet radiation, which ranges around 290 nm and 400 nm, is reaching the Earth’s surface due to the depletion of the stratospheric ozone. High doses of ultraviolet (UV) radiation affect all forms of life and its fundamental biomolecules such as proteins, lipids, and deoxyribonucleic acid (DNA) [1], directly causing cell damage by affecting the structure of DNA protein and other UV-absorbing molecules and indirectly by producing reactive oxygen species (ROS) [2]. The harmful effects that solar radiation causes are mainly due to the UV region of the electromagnetic spectrum. These can be divided into three different regions: UV-A (320 to 400 nm), UV-B (290 to 320 nm), and UV-C (200 to 290 nm) [3]. Approximately 90–99% of the solar UV radiation energy that reaches the Earth’s surface is UV-A, whereas only 1–10% is UV-B [4]. Both UV-A and UV-B are responsible for one of the biggest concerns related to UV radiation, which is skin cancer (Figure 1). UV radiation is an environmental toxic agent to which people are constantly exposed, but it does not penetrate any deeper than the skin. However, this exposure may cause epigenetic disturbances in signaling pathways causing skin cancer [5]. UV radiation-induced immunosuppression is another important event in skin cancer development [6]. UV-A radiation plays a part in immunosuppression, causing a similar number of gene mutations as UV-B in human skin cancer [7,8]. Chronic exposure to UV-A leads to a disorganization of the extracellular matrix, alteration of DNA, chronic inflammation, and alteration of fatty acids, carbohydrates, or protein structure. These alterations are related to skin aging and the increasing risk of skin cancers. The mechanisms leading to skin damage are not fully understood and may derive from the generation of ROS and subsequent lipid peroxidation [9].

To prevent skin cancer and other issues such as sunburns, photoaging, hyperpigmentation, and skin wrinkles, the use of sunscreen is recommended. The UV-B radiation that reaches the Earth is mainly responsible for these types of skin damage [10]. The most known and currently accepted method of sunscreen rating is the sun protection factor (SPF) system. However, not only the sun protection factor is a key parameter in measuring how effective a sunscreen is, but also the immune protection factor plays an important and very different role. This one correlates to the UV-A protective capability of the sunscreen for the prevention of UV immunosuppression [6]. The inhibition of lipid peroxidation (LPO) species formed due to photooxidative damage is a good measure for UV protection [11]. There are sunscreens that can decrease the LPO in the *stratum corneum*, helping to establish the efficacy of anti-UV and antioxidant agents [12]. Sunscreens should be rated against their immune protective capability, their SPF as well as their LPO inhibition to provide a better indication of their ability to protect against skin cancer and other skin diseases [6].

Being the largest organ of the body, the skin is susceptible to diseases caused by solar radiation. According to data from the World Health Organization (WHO) in 2020 there were 1.20 million new cases worldwide of non-melanoma skin cancer [13]. In countries such as the United States, the WHO has stated that one in five Americans will develop skin cancer once in their lifetime [14]. In 2020 the European Union estimated that 4% of all new cancer diagnoses corresponded to skin melanoma [15]. According to the Ministry of Health, in Mexico, there are around 1000 cases of skin cancer per year [16]. This type of cancer doubles its incidence every 10 years [16] for these reasons and the growing demand for products with sun protection factors has a significant impact on the investigation and creation of sun protectants. In Latin America, sunscreens generate sales of approximately USD 1518.8 million; in Mexico, the sales reach USD 113 million annually [17]. This indicates that the area of sunscreens is an excellent business opportunity globally, as the sunscreen market not only impacts people who want to protect themselves from skin cancer, but also people who care for their skin to protect it from sunburn and aging. The interest in consuming more environmentally friendly products has led producers of skin care products to be more responsible and to find greener alternatives when developing a product.

**Figure 2:**
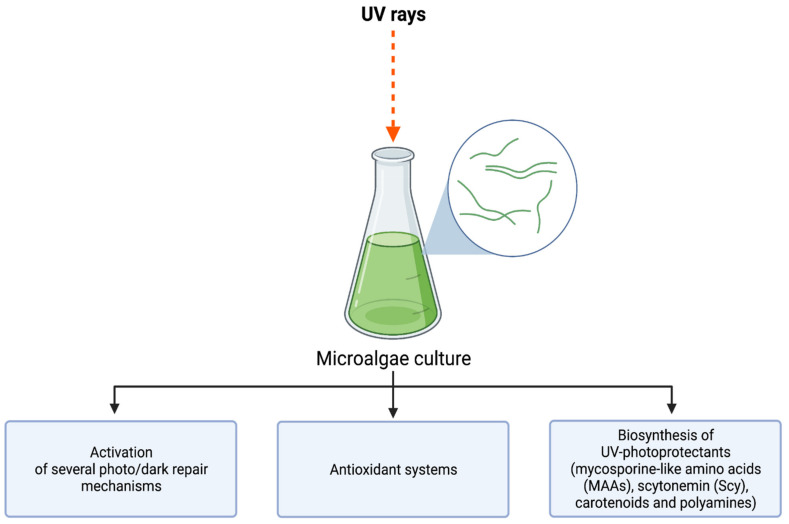
Different tolerance mechanisms against environmental UV stress present in microalgae. Created with BioRender.com (accessed on 1 May 2022) and extracted under premium membership.

To prevent photodamage, organisms have evolved to provide biochemical and physical defenses to UV by synthesizing robust bioactive compounds. For instance, phenylpropanoids and flavonoids in higher plants, melanin in animals, fungi, and microorganisms, and mycosporine-like amino acids (MAAs) in cyanobacteria, fungi, algae, and animals [18,19,20]. Some algae species synthesize substances with chemical structures that can absorb UV radiation as well as inhibit actions on melanin synthesis [19]. Studies have been performed regarding how UV radiation affects marine organisms; these studies concluded that different species of microalgae show a wide range of susceptibility to UV radiation [20]. Some marine organisms, including microalgae, have developed physiological and biochemical traits to tolerate and cope with UV rays. Microalgae can produce organic metabolites, such as sporopollenin, scytonemin, and MAAs to mention a few, and these metabolites have the purpose to protect the microalgae from UV radiation while allowing visible radiation involved in photosynthesis to pass through [21]. This characteristic allows some microalgae to be tolerant to UV rays (Figure 2). The understanding of these secondary metabolites produced by algae has led to the creation of promising industrially relevant compounds. The growing concern regarding the ecologically and dermatologically noxious implications of current sun protectants has brought with it the need for a safer alternative. MAAs are the most studied photo-protectant in marine organisms due to their relevance. They are abundant in various aquatic and terrestrial environments and are known for being eco-sustainable compounds since they are the outcome of the evolution of algae. Unlike their synthetic counterparts used unconventional sunscreens, there are no possibilities for pollution caused by them [22]. Thus, algal metabolites shape the safety and sustainability profiles of commercial sunscreens [22].

Microalgae are considered the most important biomass producers on a global scale as they have rapid growth, easy to cultivate, and can tolerate a broad range of pH, salinity, and temperature [23]. The UV radiation resistance capacity of microalgae and their potential applications in the field of sunscreens have been investigated, as they show an excellent capacity for topical application, meeting the growing demand for the creation of more environmentally friendly products.

Every year, consumers are becoming more aware of where the products they use come from, and nowadays it is not enough for a product to be effective, but whether a product is synthetic or natural, eco-friendly, or eco-sustainable matters too. Even if it is well known that sunscreens are an important tool in the fight against skin diseases, their formulations may need to be improved to contain safer ingredients, particularly in view of the concerns raised on the potential eco-toxicity of some sunscreens to the marine environment as well as to human health [24,25].

## 2. UV-Resistant Microalgae

Different species of microalgae show a wide range of susceptibility to UV-B radiation [20] and some have adapted to UV radiation over the years. Marine organisms, including eukaryotic microalgae and cyanobacteria, during evolution, have developed physiological and biochemical traits for defense and/or tolerance to UV rays [26]. Although this is an exploitable feature of microalgae, limited information is available on the molecular mechanisms involved in stress response, adaptation, and resilience of microalgae to UV radiation. Some of the research made regarding this feature is mentioned in the following section. Núñez-Pons et al. [27] reported some microalgae with UV resistant properties. For instance, *Eutreptiella* sp. from the Southern Ocean, under variable light conditions, was successfully protected by the photoprotective pigments. This research also mentioned the Antarctic microalgae *Chaetoceros dichaeta*, *Phaeocystis antarctica,* and *Polarella glacialis*, these were subjected to acclimation to photosynthetically active radiation (PAR) and showed resistance to UV-B rays. Among these Antarctic microalgae, *Polarella glacialis* was shown to possess a very high xanthophyll to chlorophyll ratio [27], which implies a high content of UV-absorbing compounds. Buma et al. support these findings stating that *P. antarctica* and *P. glacialis* showed strong linear induction of UV-absorbing compounds at increasing PAR intensities [28].

Another relevant study was conducted by Xiong et al. where species that are highly tolerant to UV-B and species that are highly susceptible are compared. It was found that the tolerant species contained substantial amounts of acetolysis-resistant residue. The residue consists of sporopollenin, a biopolymer of variable chemical composition that occurs in the algal cell walls and absorbs UV-B radiation. This study indicates that sporopollenin limits the UV-B irradiance inside the algal suspension and in individual cells by absorption caused by a mixture of aromatic components in the sporopollenin structure. With this, it was determined that sporopollenin provides protection to the tolerant species by screening the incident UV-B radiation. Previous studies showed that the MAAs act as effective UV-B screens, comparing the mechanism of action of sporopollenin and the MAAs, the first one provides constant protection while MAA are induced by radiation stress and occur with some delay [20].

So far, several species, e.g., *Coelastrella rubescens*, *Coelastrella terestris*, *Enallax coelastroides*, *Scotiella chlorelloidea*, *Ankistrodesmus spiralis*, *Chlorella minutissima*, *Chlorella sorokiniana*, and *Pseudococcomyxa* sp. have been reported as rich sources of MAAs [29]. As it was mentioned before, these act as effective UV-B screens. This report demonstrates the accumulations of MAAs when UV-B is induced. Kováčik et al. [30] states the tolerance of Scenedesmus to UV-B as well as to UV-A and UV-C. The effects of UV rays on microalgae are investigated by studying the effect of UV stress on the fatty acid and lipid class composition in two marine microalgae *Pavlova lutheri*, currently known as *Diacronema lutheri*, and *Odontella aurita* [31]. The results obtained in the investigation for *Odontella aurita* suggest that this species is more resistant and seems to be able to partially acclimate to UV rays based on the analysis of the lipids present in the microalga [31]. This finding was supported in other research [32] which mentions that the microalgae *Odontella aurita* was able to acclimate to UV rays’ stress.

More research conducted on UV-resistant microalgae includes UV-B resistance as a criterion for the selection of desert microalgae to be utilized for inoculating desert soils. The responses of photosynthetic activity, ROS generation, and DNA strand break to UV-B radiation in four microalgae isolated from artificially induced biological soil crusts were investigated. The results obtained point out different resistance and defense mechanisms of the four microalgae in response to UV-B irradiance and suggest that the strain of *Nostoc* sp. tested is the most suitable for surviving under the high UV irradiation [33]. All these findings show the proven resistance of some microalgae, but also demonstrate the susceptibility of some others. This shows that more research must be completed to determine the resistance of more microalgae; however, this research has established the initial step for a new opportunity in the production of sunscreens based on these compounds that confer resistance to UV rays. Table 1 shows a summary of the findings on some of the most relevant UV-resistant microalgae along with their resistance factor and the experimental conditions to which they were exposed [34,35,36,37,38,39,40,41,42,43,44,45,46,47].

## 3. UV-Induced Microalgae Biosynthesis

In aquatic environments, where microalgae are prominent, the presence and role of UV-absorbing compounds have been the subject of study in recent years. Certain microalgae produce organic metabolites, such as sporopollenin, scytonemin, and MAAs as well as other UV-absorbing substances to counteract the harmful effects of UV radiation and protect themselves while allowing visible radiation involved in photosynthesis to pass through [21,48]. The best known photoprotective response in marine organisms is the production or accumulation of UV-absorbing compounds, including MAAs as the most common compounds with such properties, but there are also others, such as scytonemin, carotenoids, alganean, and various secondary metabolites [27].

### 3.1. Mycosporine-Like Amino Acids (MAAs)

MAAs are small intracellular, colorless water-soluble secondary metabolites of low molecular weight, commonly found in marine environments. These compounds have absorption maxima in the UV region between 310 and 360 nm, they can dissipate radiation as heat without producing ROS. Under fixed light, MAA biosynthesis seems the most effective photoprotective mechanism [45]. More specifically, the biosynthesis of MAAs happens principally via the shikimate pathway [51,52,53,54,55]. MAAs have been found in tropical to Antarctic waters and in a variety of organisms, spanning from cyanobacteria, microalgae, fungi, as well as macroalgae, and animals. In addition to protecting cells from mutation caused by UV rays and free radicals, they are also effective antioxidant molecules [27].

More than thirty different MAAs have been identified from marine and terrestrial environments [56]. The vital roles of different MAAs in protecting mechanisms by virtue of their UV-absorbing and antioxidant function have been reported in several microalgae [26]. MAAs are considered the best photoprotective compound in microalgae due to their strong absorption capability in the UV spectrum, photostability in both fresh and saline water in the presence of photosensitizers, their antioxidant properties, and resistance to several abiotic stressors such as temperature, UV radiation, and various solvents and pH. For these reasons, they potentially can prevent UV-induced skin damage [57]. It has been reported that MAAs protect against DNA damage, effectively block thymine dimer formation by UV radiation, protect the cells from UV-induced cell death, they also promote the proliferation of human skin fibroblast cells [58]. These characteristics make MAAs an excellent alternative for green biotech applications in sunscreens.

### 3.2. Carotenoids

Carotenoids are structurally and functionally very diverse natural pigments and important components of the photosynthetic apparatus, they enhance cellular photosynthetic production and provide photooxidative protection. One of the most characteristic features of carotenoids is their strong coloration, and this characteristic is crucial for the proper functioning of light absorption in photosynthetic organisms and photoprotection in all living organisms. This feature plays an important role in photoprotection against UV-A radiation in cyanobacteria. Several carotenoids such as canthaxanthin, echinenone, myxoxanthophyll, and zeaxanthin with protective functions against photo-oxidative damage have been reported in several cyanobacteria and other green algae species, e.g., *Coelastrella rubescens* [42]. Nearly all carotenoids absorb light in the 400–500 nm range. Their quenching properties allow the dissipation of excess energy from UV-B [27]. Carotenoids play an important role in absorbing light energy for use in photosynthesis as well as protecting chlorophyll from being damaged by visible or UV light. The content of total carotenoids has been found to correlate with their protection against UV light [25].

These carotenoids accumulate in microalgae under stress conditions such as high-light and UVA radiation suggesting a synergy effect with MAAs providing a range from UVA to the blue–green spectrum of attenuation [42].

### 3.3. Sporopollenin

Sporopollenin is an acetolysis-resistant inert biopolymer possessing a complicated structure with aliphatic (mainly isoprenoid) and aromatic components variably present. It is found in the cell wall of some algae and in plant pollens and spores [21]. It was reported that species of microalgae that were highly tolerant to UV radiation had substantial amounts of sporopollenin. This compound functions as an antimicrobial agent, confers rigidness to the cell wall, and may further protect from UV rays by increasing the optical density, whereas species containing little or no sporopollenin were highly UV radiation susceptible. In the pollen and spores of Antarctic plants, sporopollenin is considered a bio-indicator of solar UV-B and a valuable archive for the reconstruction of past solar UV-B [20]. Sporopollenin-like substances seem to be the key protectors of some microalgae against UV. Due to high stability, sporopollenin-like compounds provide constant UV protection [42].

### 3.4. Scytonemin

Scytonemin is a yellow-brown lipid soluble sheath pigment that absorbs maximally in the UV-A and UV-C regions, but with some absorbance in the UV-B region. It has an in vivo absorption maximum of 370 nm. It is synthesized by both temperature increase, and oxidative stress combined with UV-A [27]. Other reports determine that scytonemin is suspected to be synthesized from metabolites of aromatic amino acid biosynthesis [21]. Scytonemin is located in the extracellular polysaccharide sheath of some cyanobacteria acting as a passive sunscreen against UV radiation. It can also reduce about 90% of solar UV-A radiation that can reach the cell. Microalgal scytonemin appears restricted to cyanobacteria. The high stability of this compound against stressors such as temperature and UV-A radiation and its capability to undertake UV screening even after prolonged physiological inactivity such as desiccation makes it a strong candidate for use as a natural UV screening compound for humans [21].

### 3.5. Phenolic Compounds

Phenolic compounds, which are also present in microalgae, also likely contribute to UV tolerance. Phenolic compounds in brown algae contribute considerably to protecting sensitive life stages from irradiation [59]. These compounds are one of the largest groups of secondary metabolites present in plants, ranging from simple aromatic rings to more complex molecules and comprising flavonoids, phenolic acids, tannins, lignans, or coumarins. Phenolic compounds are also a part of the UV tolerance sector of interest, they are used in cosmetics as UV-protection and antioxidant agents [60].

## 4. Bio-Carriers for Skin Applications

Controlled drug delivery is one of the most important areas of research in the creation of skin care products such as cosmeceuticals. It is desired for most drugs due to safety and efficacy reasons. By encapsulating such drugs into biodegradable polysaccharide materials, both improved bioavailability and controlled drug release is expected [61]. Bio-based materials as bio-carriers are noted as key ingredients for the development of drug delivery systems [62], while polysaccharides are the basis of the production of bio-based materials [63]. However, due to their properties such as bioavailability, biodegradability, stability, availability, renewability, and low toxicity, polysaccharides can be part of pharmaceutical products, but they can also be applied as independent solid matrices in different forms (monoliths, beads, micro- or nanoparticles). Nanoparticles based on hydrophilic polymers are appropriate candidates for drug delivery [64].

A good product formulation allows the user to obtain the greatest possible benefits from the product to be used. Different bio-carriers will perform different functions, some bio-carriers used in cosmetics that can be used for the formulation of sunscreen gels for their drug delivery potential are alginates, chitosan, gelatin, and cellulose to mention a few. Table 2 shows a summary of some microalgae used along with bio-carriers for drug delivery [65,66,67,68,69,70,71,72,73].

Alginate is a hydrophilic polysaccharide extracted from marine brown algae such as *Laminaria hyperborea* or soil bacteria such as *Azobacter vinelandii* [74]. It has often been used in medical applications such as wound dressings, scaffolds for hepatocyte culture and surgical or dental impression materials. Alginate is commonly used in the food industry to modify food properties such as rheology (thickening), water binding capacity, stabilizing emulsion, and film formation. This compound is able to break down to simpler glucose type residues and be totally absorbed as well as to gel by ionic crosslinking [75]. Hydration of alginic acid, a compound present in alginates, at low pH, leads to the formation of a high viscosity “acid gel” due to intermolecular binding; this gelatinous layer formed can act as a drug diffusion barrier [76]. Alginate gels are stable in the temperature range 0–100 °C. After gelation, the water molecules are physically entrapped inside the alginate matrix but are still free to migrate. This application is relevant for the creation of alginate gels for cell immobilization/encapsulation [77]. This application of alginate as a delivery system has been used in the microalgae field. Some of these applications have been in the creation of a 3D matrix with cellulose derivatives combined with a crosslinking agent, the alginate, to obtain a matrix capable of retaining and releasing bioactive components [70].

Chitosan is the deacetylated form of chitin, it is a linear polymer widely studied for being nontoxic, biodegradable, non-immunogenic, antibacterial, and a biocompatible polymer [64]. The viable bioactivity of chitosan has made it an interesting biomaterial applying it in hematology, immunology, wound healing, drug carrying and cosmetics [78]. Chitosan-based matrices have been widely used in the biomedical field for cell encapsulation, drug delivery, cell culture, hyaline cartilage repair and bone reconstruction. Hydrogels of chitosan have been used before to induce an appropriate response for the reconstruction of the skin after a third-degree burn injury, on a limited area [79]. Additionally, this polymer is hydro-soluble, which makes it ideal for drug delivery. All these features facilitate the uptake of a drug through the cell membrane [80,81,82].

Gelatin is derived from collagen, which is a ubiquitous fibrous protein found extensively in the tissues of mammalian species. Gelatin is practically more convenient than commercially used collagen because a concentrated collagen solution is extremely difficult to prepare from native collagen and furthermore gelatin is far more economical than collagen [78]. Gelatin is applied as a gelling ingredient in many cosmetic products including face creams, body lotions, shampoos, hair sprays, sunscreens, and bath salts and bubbles. Fish gelatin hydrolysates (proteins and peptides) were used to prevent the damage caused by UV radiation on the skin. Another mechanism of gelatin and its hydrolysates in the protection against UV radiation is boosting immunity by elevating the thymus index and boosting the immune increasing hydroxyproline in the skin, which is an indicator of the collagen content. Similarly, gelatin hydrolysates obtained from fish gelatin of tilapia (*Oreochromis niloticus*) were found to have a scavenging effect against ROS of UV that causes damage to the skin and results in early aging [83].

Cellulose is one of the most abundant biodegradable materials in nature and has thus been the topic of extensive investigations in macromolecular chemistry. Developments in molecular biology and the application of cell systems in vitro have resulted in extensive exploration of the mechanisms underlying the biosynthesis of cellulose in nature. Cellulose-based polymers have wide applications in tissue engineering, controllable delivery system, blood purification, sensor, agriculture, as well as water purification. Bacterial cellulose was shown to have great potential for biomedical application and very high clinical value for skin tissue repair [84].

## 5. Conclusions, Challenges, and Future Considerations

Microalgae are constantly exposed to UV rays because they are usually found in areas with high sunlight exposure. Several studies have addressed the harmful impact of increased UV radiation on various microalgae. However, some microalgae have a great ability to overcome or counteract different stresses such as intense solar UV radiation, this has been happening over the years and with the evolution of microalgae, some examples of defense and/or tolerance mechanisms are: DNA repair, synthesis of antioxidants, and enzymatic/non-enzymatic compounds, such as MAAs and scytonemin to counteract the harmful effects of UV radiation. This characteristic has not only been investigated to learn more about microalgae, but also for applications such as product creation, since they grow rapidly and are considered the most promising and sustainable sources of biomass. Microalgae have shown a promising future in different areas of industry such as agriculture, energy, and cosmetics. Due to their performance under optimal conditions, they have been widely studied for different bioproducts. Bio-carriers can provide added value for the development of drug delivery systems in different microalgae bioproducts due to their benefits such as bioavailability, biodegradability, stability, availability, renewability, and low toxicity. However, the potential of microalgae as topical photoprotectants has not been fully exploited, since there are not a lot of studies regarding this application. On the other hand, inorganic compounds and their interaction with microalgae derived compounds were not abording in this work. Nevertheless, it is important to consider possible interactions in a formulation, to improve protective effects or to avoid the degradation or inhibition of microalgae compounds protecting effects. Still, it is undeniable that there is a very strong potential for microalgae to be used in the UV skin care industry as they can protect from UV-A, UV-B and in some cases even UV-C. Both UV-B and UV-A rays present negative effects in the short and long term, which is why microalgae extracts, by showing resistance to both types of rays, show great potential as sunscreens and this gives a very strong advantage to future bioproducts.

## Figures and Tables

**Table 1:** Microalgae that have been reported to have UV resistance compounds.

Microalgae	UV Type	Units/Range	Resistance Factor	Type of Study	Culture Media	Growth Temperature	Reference
*Anabaena* spp. *Leptolyngbya* sp.	UV-B	1 W m^−2^ for 4 h/day	Scytonemin, MAAs	Exposure to UV-B radiation	BG-11 (without nitrogen sources) with NH4Cl	28 ± 2 °C	[35,36]
*Arthrospira platensis*	UV-A	320 nm	Sporopollenin, Scytonemin, MAAs	Exposure to UV-A irradiance	BG-11 medium	23 °C	[36]
*Botryococcus braunii*	UV-B	250 to 450 nm	Self-emulsifying base cetearyl alcohol (and) dicetyl hosphate (and) ceteth-10 phosphate (Crodafos^®^ CES)	Analysis of photoprotection activity	CHU medium	25 ± 1 °C	[37]
*Haematococcus rubicundus, Bracteacoccus aggregatus,* and *Deasonia* sp.	UV-A	250 to 400 nm	MAAs, Carotenoids	Exposure to UV-A radiation	BG-11	25 °C	[38]
*Chaetoceros dichaeta*	UV-B	400 to 700 nm	Antioxidant enzyme superoxide dismutase	Acclimation to PAR	GP5 medium	1.0 ± 0.5 °C	[27,28]
*Synechococcus* sp. PCC7942 (*Cyanophyta*), *Synechocystis* sp. *PCC6803* (*Cyanophyta*), *Chlorella protothecoides* (*Chlorophyta*), *Chlamydomonas reinhardtii* (*Chlorophyta*), *Phaeodactylum tricornutum* (*Bacillariophyta*), *Alexandrium tamarense* (*Pyrrhophyta*) and *Dicrateria zhanjiangensis* (*Chrysophyta*)	UV-C	0.01 to 0.20 W m^−2^	Chlorophyll a (Chl a), Carotenoids	Exposure to different UV-C irradiances	BG-11 medium	25 ± 1 °C	[40]
*Chlorella vulgaris*, *Chlorococcum citriforme*	UV-B	1 to 5 W m^−2^	Sporopollenin, Scytonemin, MAAs	Exposure to UV-B radiation	Bold Basal medium	25 ± 1 °C	[41]
*Coelastrella rubescens*	UV-A	380 to 415 nm, power of 2.9 W m^−2^	Carotenoids, MAAs, Sporopollenin	Exposure to high fluxes of visible light and UV-A	BG-11 medium	24 °C	[42]
*Dunaliella* spp. (*Dunaliella bardawil*, and *Dunaliella salina*)	UV-B, UV-A	110 mmol m^−2^s^−1^ for UV-A	β-carotene	Exposure to UV-A and UV-B radiation	Medium with NaCl	26 °C	[43]
*Dunaliella* spp.	NR	2 W m^−2^	MAAs	Long-term exposure to artificial UV radiation	NR	NR	[44]
*Scenedesmus* sp.	UV-B	2 to 15 W m^−2^	MAAs (mycosporine-Gly, palythine, asterina, shinorine and porphyra)	Exposure to extreme UV-B irradiance	NR	26 °C	[29]
*Eutreptiella* sp.	UV-B	250 to 750 nm	Xanthophylls, MAAs	Tested under fixed light	f/2 medium	10 °C	[45]
*Halamphora coffeaeformis*	UV-B, UV-A	NR	Recovery of the photosynthetic parameters	Exposure to UV-A and UV-B radiation	Artificial seawater	NR	[32]
*Haematococcus lacustris*	NR	500 lux	Astaxanthin	Exposure to artificial UV radiation	Bold Basal medium	24 °C	[34]
*Leptolyngbya tenuis*	UV-B	0.8 ± 0.1 mW cm^−2^	Scytonemin, MAAs	Exposure to UVB-R	ASN III medium	30 ± 2 °C	[34,46]
*Lyngbya* sp.	UV-B	8 W m^−2^	MAAs, Scytonemin	Exposure to UV radiation	Liquid culture medium	23 °C	[35,47,48]
*Nannochloropsis oculata*	UV-A	6 to 24 W m^−2^	Sporopollenin, Scytonemin, mycosporine-like amino acids	Exposure to different levels of UVA radiation	Instant Ocean artificial sea water	25 °C	[34,49]
*Nostoc* sp.	UV-B	312 nm	Carotenoids, Scytonemin	Analysis of photosynthetic activity	BG-11 medium	25 °C	[27,33]
*Nostoc sphaeroides*	UV-A	320 nm	Not identified	Exposure to UVA irradiance	BG-11 medium	23 °C	[50]
*Odontella aurita*	UV-B, UV-A	110 kJ m^−2^	D1 protein, Activation of antioxidant enzymes	Exposure to UVA-R and UVB-R	Artificial seawater	NR	[31,32]

NR: Not reported; MAAs: mycosporine-like amino acids.

**Table 2:** Applications of bio-carriers with microalgae for drug delivery.

Material Used	Microalgae	Component Loaded	Formulation	Purpose	Types of Tests	References
Chitosan	*Spirulina*	5-Fluorouracil	Chitosan (1%(*v*/*v*)), *Spirulina* microalgae and genipin.	Drug delivery	In vitro cytotoxicity test	[65]
Alginate and Chitosan	*Auxonochlorella* *protothecoides*	Microalgae oil extract	PVA solution (7–8% (*w*/*v*)) with sodium alginate (2% (*w*/*v*)) and PVA solution (7–8% (*w*/*v*)) with chitosan (2–3% (*w*/*v*))	Nanoparticle production to deliver bioactive compounds in microalgae	In vitro release testing	[66,67,68]
Alginate, PVA	*Botryococcus braunii* and *Microcystis aeruginosa*	Microalgae oil extract	PVA solution (8% (*w*/*v*)) and sodium alginate (2% (*w*/*v*))	Nanoparticle production to deliver bioactive compounds in microalgae	In vitro release profile of nanoparticles	[66,69]
Alginate, cellulose	*Chlorella vulgaris*	Tea tree essential oil	Microfibrillated cellulose, nanofibrillated cellulose and carboxymethylcellulose (0.01% (*m*/*v*)) with alginate (2% (*m*/*v*))	Cosmetic application	Differential scanning calorimetry	[70]
Cellulose	*Chlorella pyrenoidosa*	-	Microalgae powder, sodium dodecyl sulfate	Sensor for pollutant detection	-	[71]
Gelatin	*Phaeodactylum tricornutum*	Microalgae extract	*P. tricornutum* powder (0.5% or 1.0% (*w*/*v*)), gelatin solution	Wound dressing with antimicrobial *P. tricornutum*-loaded gelatin nanofiber mat	In vitro studies for antibacterial activity	[72,73]

## Data Availability

Not applicable.
